# The Use of Biomonitoring Data in Exposure and Human Health Risk Assessments

**DOI:** 10.1289/ehp.9056

**Published:** 2006-06-12

**Authors:** Richard Albertini, Michael Bird, Nancy Doerrer, Larry Needham, Steven Robison, Linda Sheldon, Harold Zenick

**Affiliations:** 1 University of Vermont College of Medicine, Burlington, Vermont, USA; 2 ExxonMobil Biomedical Sciences Inc., Annandale, New Jersey, USA; 3 International Life Sciences Institute, Health and Environmental Sciences Institute, Washington, DC, USA; 4 Centers for Disease Control and Prevention, Atlanta, Georgia, USA; 5 The Procter & Gamble Company, Cincinnati, Ohio, USA; 6 National Exposure Research Laboratory, U.S. Environmental Protection Agency, Research Triangle Park, North Carolina, USA; 7 National Health and Environmental Effects Research Laboratory, U.S. Environmental Protection Agency, Research Triangle Park, North Carolina, USA

**Keywords:** arsenic, biomarkers, biomonitoring, exposure, methyl eugenol, organophosphorus, PBDE, PFOS, phthalates, risk assessment

## Abstract

Biomonitoring uses analytic methods that permit the accurate measurement of low levels of environmental chemicals in human tissues. However, depending on the intended use, biomonitoring, like all exposure tools, may not be a stand-alone exposure assessment tool for some of its environmental public health uses. Although biomonitoring data demonstrate that many environmental chemicals are absorbed in human tissues, uncertainty exists regarding if and at what concentrations many of these chemicals cause adverse health outcomes. Moreover, without exposure pathway information, it is difficult to relate biomonitoring results to sources and routes of exposure and develop effective health risk management strategies. In September 2004, the Health and Environmental Sciences Institute, U.S. Environmental Protection Agency, Centers for Disease Control and Prevention, Agency for Toxic Substances and Disease Registry, and International Council of Chemical Associations co-sponsored the International Biomonitoring Workshop, which explored the processes and information needed for placing biomonitoring data into perspective for risk assessment purposes, with special emphasis on integrating biomarker measurements of exposure, internal dose, and potential health outcome. Scientists from international governments, academia, and industry recommended criteria for applying biomonitoring data for various uses. Six case studies, which are part of this mini-monograph, were examined: inorganic arsenic, methyl eugenol, organophosphorus pesticides, perfluorooctanesulfonate, phthalates, and polybrominated diphenyl ethers. Based on the workshop and follow-up discussions, this overview article summarizes lessons learned, identifies data gaps, outlines research needs, and offers guidance for designing and conducting biomonitoring studies, as well as interpreting biomonitoring data in the context of risk assessment and risk management.

In this mini-monograph, “biomonitoring” is the analytical measurement of biomarkers in specified units of tissues or body products (blood, urine, etc.). These biomarkers are any substances, structures, or processes so measured that indicate an exposure or susceptibility or that predict the incidence or outcome of disease (Toniolo et al. 1997).

Public and private demands for biomonitoring data are on the increase. In the United States, government-sponsored programs include the National Health and Nutrition Examination Survey (NHANES) conducted by the Centers for Disease Control and Prevention (CDC 2001, 2003, 2005), the CDC National Environmental Public Health Tracking Program (CDC 2004), the inter-agency National Children’s Study (NCS 2005), the interagency Agricultural Health Study (AHS 2005), the Farm Family Exposure Study (University of Minnesota 2004), and the pilot studies conducted by the U.S. Environmental Protection Agency (U.S. EPA) as part of the National Human Exposure Assessment Survey (NHEXAS) (U.S. EPA 2004). The National Institute of Environmental Health Sciences (NIEHS) and the U.S. EPA currently sponsor research to identify relevant environmental exposures in 12 Centers for Children’s Environmental Health and Disease Prevention Research around the United States (NIEHS 2003). The National Academy of Sciences/National Research Council Committee on Human Biomonitoring for Environmental Toxicants has reviewed “current practices and recommends ways to improve the interpretation and uses of human biomonitoring data for environmental toxicants” (NRC 2004). Epidemiology studies, which include a biomonitoring component, are also under way in numerous academic institutions.

In Europe, the European Commission (EC) has developed background materials for environmental health, named SCALE (Scientific evidence, focused on Children, meant to raise Awareness, improve the situation by use of Legal instruments, and ensure a continual Evaluation of the progress made) (EC 2004a). SCALE was followed by the European Union’s (EU) environmental health action plan, with “Action 3” focusing on biomonitoring. A specific support action program to prepare a European biomonitoring pilot study in 2007 has been initiated (EC 2004b). The EU has financed several research programs that focus on development and validation of biomarkers.

The European Centre for Ecotoxicology and Toxicology of Chemicals (ECETOC) published “Guidance for the Interpretation of Biomonitoring Data” (ECETOC 2005), which reviews the purpose and uses of biomonitoring data, offers guidance on interpretation, and suggests a framework for placing biomarker data into context. Also, breast milk monitoring programs provide surveillance data for characterizing high-risk population exposures. So important is the collection of biomonitoring data that several prominent European Union officials in 2004 gave blood as part of a World Wildlife Fund (WWF) survey to demonstrate the presence of environmental chemicals in the human body (WWF 2004). Although these cited examples are by no means a complete inventory of existing or planned biomonitoring activities, they provide the reader with a snapshot of the magnitude of global interest in biomonitoring.

The flurry of activity associated with biomonitoring data collection reflects, in part, the risk assessment community’s call for more and better exposure data, especially in the context of population-based studies. Now, risk assessors face the difficult challenge of how best to interpret and apply biomonitoring data. Although the availability of human biomonitoring data for many chemicals can greatly reduce the uncertainty associated with the use of external exposure estimates in risk assessment, experience and guidance are needed to integrate the use of biomonitoring data into the risk assessment process.

Biomonitoring is not a new phenomenon. For more than a century, occupational physicians and industrial hygienists have monitored worker populations for exposure to a variety of hazardous substances. Clinical medicine offers historical and contemporary lessons on the value of measuring human body fluids for indicators of adverse health risk (Sexton et al. 2004). Ideally, biomonitoring data from environmental, occupational, and clinical settings are supported by quality control, analytical standardization, availability of control groups, and other mechanisms for limiting uncertainty and variability. Biomonitoring programs for assessing exposure to environmental chemicals generally require the measurement of the relevant analytes at much lower concentrations than needed in human clinical or animal toxicology studies, thus posing considerable challenges. Therefore, biomonitoring approaches for assessing exposures to environmental chemicals must employ state-of-the-art analytical methods, which often include isotope-dilution mass spectrometry, to limit the uncertainty for measuring low-level concentrations. Improved analytical capabilities make possible the accurate and precise measurement of many environmental chemicals at very low levels in the tissues of the general population, thus demonstrating human exposure to and absorption of chemicals, and often their distribution, metabolism, storage, and elimination.

On 21–22 September 2004 the International Life Sciences Institute’s (ILSI) Health and Environmental Sciences Institute (HESI) organized and co-sponsored the International Biomonitoring Workshop with the U.S. EPA National Exposure Research Laboratory, the CDC National Center for Environmental Health, the Agency for Toxic Substances and Disease Registry, and the International Council of Chemical Associations. The workshop was held at the laboratory facilities of the U.S. EPA in Research Triangle Park, North Carolina.

At the workshop, key questions relating to the use of biomonitoring data in the context of risk assessment were discussed: How can biomonitoring data be used? What other information is needed to apply biomonitoring data in a risk assessment context? How might biomonitoring data be used inappropriately? To explore these questions, case studies were prepared in advance and discussed in small working groups. The case studies included chemicals that are short-lived in the environment and readily metabolized in humans, as well as chemicals that are relatively stable in the environment and bioaccumulate. Some case studies were data rich and were compared and contrasted with chemicals for which few data exist. This mini-monograph includes six case studies examined at the workshop. In evaluating the case studies, workshop participants identified both general and specific scientific issues, questions, and research needs.

The environmental public health continuum (EPHC; Figure 1) served as an important starting point for the 100 workshop invitees from international governments, academia, and industry. The workshop focused on the links between the components of the EPHC as well as on the components themselves. The unique utility of the EPHC as a tool is that one may start at any point on the continuum and work forward or backward through the links. Because the links work both ways, it is possible to examine what is not known and which data gaps need to be filled. Careful definition of the link being assessed, as well as the question being asked, is critical.

At the International Biomonitoring Workshop, participants explored the processes and information needed to place biomonitoring data into perspective for the risk assessment process, with special emphasis on integrating biomarker measurements of exposure, internal dose, and potential health outcome. The EPHC was modified to capture the critical workshop focus area (Figure 2). The reliance on and integration of hazard identification with exposure and dose are recognized in this diagram.

One outcome of the workshop is the development of guidance on the application of biomonitoring data in the context of risk assessment, risk management, and disease prevention. Different criteria are recommended for applying and interpreting biomonitoring information for different purposes. For instance, epidemiology/human effects data would not be needed to address the question of whether there is a trend for a substance or an increase (or decrease) in the environment over time; however, epidemiology/human effects data (and or animal toxicology data) would be necessary if the question being asked is whether there is a potential human health risk from exposure to the substance. The risk assessor/risk manager will use different data and criteria depending on the question (Doerrer and Holsapple 2004). The guidance, criteria, and questions outlined here are a general starting point for designing and conducting biomonitoring studies, as well as interpreting biomonitoring data in the context of risk assessment and risk management.

## Analytical Methods/Biomarkers of Exposure

The case studies reviewed in this mini-monograph address the ability of the analytical methods to measure the analytes of interest for the stated biomonitoring purposes:

- For phthalates, the ubiquitous occurrence of this chemical class necessitated a focus on their metabolites, specifically the monoesters—the presumed toxicologically relevant form of the chemical (Calafat and McKee 2006). For purposes of this mini-monograph, di(2-ethylhexyl) phthalate (DEHP) and diethyl phthalate (DEP) were examined as case studies. It should be noted that, in the case of DEHP, additional metabolites, which have higher urinary concentrations and thus are more sensitive indicators of exposure to DEHP than the monoester, can also be measured.
- For methyl eugenol, the parent compound can be measured in serum, which is an indication of exposure. However, the toxicologically relevant metabolite may be the DNA-reactive 1′-hydroxy species (Robison and Barr 2006).
- For arsenic, it is important to identify whether organic or inorganic forms are of primary interest, as well as the valence state (III or V) (Hughes 2006).
- Analytical methods for measuring perfluorooctanesulfonate (PFOS) in serum have been established and are undergoing further refinement (Butenhoff et al. 2006). Based on a growing body of literature, there is evidence that PFOS distributes mainly in blood and liver, where it is bound to protein, is not readily metabolized, and has an elimination half-life of several years in humans.
- The organophosphorus pesticides are rapidly metabolized and excreted in the urine (Barr and Angerer 2006). Many methods have been used for measuring the common metabolites of malathion and chlorpyrifos, the two organophosphorus pesticides examined in this mini-monograph. In most instances, the comparability of these methods has not been established, although analytical techniques using high-performance liquid chromatography interfaced with tandem mass spectrometry provide the most reliable data. In addition, many of the organophosphorus metabolites measured can be derived from environmental exposures to the preformed metabolite itself, complicating interpretation of the biomonitoring data (Barr and Angerer 2006).
- The polybrominated diphenyl ethers (PBDEs) are ubiquitous in the environment, and there is evidence that some of their congeners bioaccumulate. The PBDEs are normally found as a mixture of congeners, but specific congeners can be measured when assessing exposure to the commercial products containing the penta-and octabromodiphenyl ethers (Birnbaum and Cohen Hubal 2006).

As noted, the selection of the proper metabolites for the biomonitoring program is important. Similarly, the performance of the laboratory cannot be overstated. Every biomonitoring laboratory should participate in inspections such as those conducted in the United States according to the Clinical Laboratory Improvement Amendments (CLIA 1988) and participate where available in interlaboratory studies. One such demonstration of needs of interlaboratory comparison data is given in the inorganic arsenic case study (Hughes 2006). Reference standards and human urine samples were spiked with different amounts of the same species of arsenic and analyzed by different laboratories. When the arsenic levels in the urine samples were at concentrations that are relevant to research on the metabolism of arsenic in humans (> 5 μg/L), the variance between laboratories was low. However, when arsenic levels were lower (< 5 μg/L), there was considerably more variation, especially in methods for which the detection limits were in the range of 1–5 μg/L. Clearly, this indicates poor interlaboratory comparison at lower concentrations that is, in large part, due to differences in analytical methods.

The strategy used to collect samples for biomonitoring needs to be carefully developed. For example, in the inorganic arsenic case study, sampling strategy was considered, but historical experience indicates that there is relatively little intraindividual variability in urinary arsenic levels (Hughes 2006). Thus, sampling time may be less of a consideration for an individual. However, because of potential problems with arsenic in seafood, which can confound exposure results based only on total arsenic urinary analysis, subjects should refrain from consuming seafood for a few days before urine collection. Also, there may be differences in the metabolism and excretion of inorganic arsenic between children and adults (Concha et al. 1998). Thus, consideration of age of the population studied may be important for arsenic exposure analysis. By contrast, methyl eugenol is rapidly absorbed from the gastrointestinal tract and metabolized to multiple species, including hydroxy acids, *O*-demethylation, and hydroxylation of the benzene ring (Robison and Barr 2006). In one human uptake study, sampling was conducted at fixed time points after ingestion of cookies containing methyl eugenol (Schecter et al. 2004). The results of this study indicate that serum methyl eugenol concentrations peak approximately 1 hr after an acute exposure and are close to pre-exposure or background levels approximately 2 hr after ingestion. Because methyl eugenol is a naturally occurring chemical found in spices and herbs, such as allspice and basil, any biomonitoring study should also include consideration of dietary habits. In the case of the human uptake study, fasted subjects were used, which greatly reduced the potential variability caused from concurrent exposures to methyl eugenol through the normal diet. Nevertheless, existing background levels, albeit low, suggest that some portion of the methyl eugenol resides in a third compartment, potentially adipose tissue, which is in equilibrium with its blood concentration.

The phthalates case study highlights an additional consideration for sampling strategy, that is, knowledge about potential sources of sample contamination (Calafat and McKee 2006). Because DEP and DEHP are relatively ubiquitous materials that can be found in plastics (DEHP) and soaps/cleaning solutions (DEP), laboratory containers and cleaning solutions that contain fragrances are potential sources of contamination. As illustrated in the case study, a straightforward solution to this sampling problem was to measure phthalate metabolites in urine rather than the parent compounds.

Given the lessons learned from the case studies, several key questions regarding analytical approaches for biomonitoring should be considered:

- Were standard reference materials used that were prepared in the biologic matrix of interest (matrix based)?
- What are the specificity and sensitivity of the analytical method?
- Is the biomarker of exposure valid for the intended use (i.e., Does it accurately reflect the intended use?) [Validity is defined as the (relative) lack of systematic measurement error when comparing the actual observation with a standard. Validity differs from reliability in that reliability is the extent to which an experiment or measurement procedure yields the same results (tendency toward consistency) on repeated trials.]
- Have there been intra- or interlaboratory comparisons of methods?
- Did the sampling strategy include consideration of toxicokinetics?
- Did the sampling strategy include consideration of potential sources of error or sample contamination?
- Did the sampling strategy include consideration of the stability of the compound in question with respect to the appropriateness of sample collection and storage methods?

## Exposure

Biomonitoring data represent an integration of exposure from all sources and routes, which provides an important perspective on overall exposure. Collection of serial biomonitoring samples over an extended period of time can provide information regarding variability and trends in exposure. Such information is particularly useful for assessing the effectiveness of environmental remediation programs or evaluating the impact of removal or reduction in general use of a chemical (e.g., lead). However, the primary sources of exposure for many of the case study chemicals discussed in this mini-monograph are not fully understood. For example, in the case of DEP, it is known that fragranced cosmetic and other consumer products may contain DEP; however, the use of DEP in the cosmetic and fragrance industry accounts for < 20% of all DEP production (Api 2001). Consequently, many other sources of exposure are likely to contribute to the human DEP body burden. Potential sources of exposure to DEHP are numerous (e.g., medical plastics such as tubing and syringes; household materials such as floor or wall coverings, and plastic toys) (Calafat and McKee 2006).

Organophosphorus pesticides are used widely in agriculture and to a lesser extent in residential applications. Because organophosphorus pesticide residues have been detected at permissible (and sometimes impermissible) levels in many agricultural products, low-level dietary exposures to organophosphorus pesticides are likely. Other potential sources of exposure to a few organophosphorus pesticides still registered for residential uses include pre-construction termite control and home and garden use. In general, occupational exposures to organophosphorus pesticides dwarf environmental exposures (Barr et al. 2004, 2005); however, special populations, such as farm-worker children, may receive higher exposures. General population exposures based on biomonitoring data appear low whether or not the contribution from exposures to preformed metabolites is considered.

PBDEs and PFOS are ubiquitous in the environment and have been detected in various human biologic samples. PBDEs are found in hard plastics, electronics, textiles, and polyurethane foam products. Past or current commercial uses of PFOS predominantly include surface treatments for soil- and stain-resistant coating on fabrics, carpets, and leather; coatings on paper and packaging products for grease and oil resistance, including food contact papers; and performance chemical uses, such as fire-extinguishing foam concentrates, mining and oil surfactants, electroplating and etching bath surfactants, household additives, chemical intermediates, coatings and coating additives, carpet spot cleaners, and insecticide raw materials. Although the uses of PBDEs and PFOS have been identified, little is known about the sources of exposure and how PBDEs and PFOS enter the environment (Birnbaum and Cohen Hubal 2006; Butenhoff et al. 2006). Although both PBDEs and PFOS have been used in various consumer products, neither of these classes of chemicals was expected to be found in measurable levels in human tissues, given their specific chemical properties and uses. Environmental and ecologic monitoring data such as dust levels, market basket surveys, and fish and wildlife surveys can help to identify sources of exposure and shed light on where research should be focused.

In the case of PFOS, significant geographic differences in human concentrations indicate that different exposures have occurred (Butenhoff et al. 2006). North Americans, particularly those in the southeastern United States, are generally more exposed than are Europeans or Asians. Sex-related differences have been reported inconsistently in U.S. populations. Japanese studies have reported higher PFOS concentrations in men. As discussed in Butenhoff et al. (2006), the mechanisms and pathways leading to the presence of PFOS in human blood are not well characterized but likely involve exposure to environmental PFOS or precursor molecules and residual levels of PFOS or PFOS precursors in industrial and commercial products. This highlights the need to develop a better understanding of the potential for these compounds to persist in the environment, as well as their environmental fate. Based on the biomonitoring data available for PFOS and PBDEs in the United States, the range of biologic values in the general population is quite large, and the data are log-normally distributed (Birnbaum and Cohen Hubal 2006; Butenhoff et al. 2006).

Arsenic is an example of a chemical that occurs naturally in the environment and is also introduced as a result of human activity. This further illustrates the need for a detailed understanding of potential sources of exposure and how those sources contribute to overall exposure. In addition, multiple species of arsenic must be considered potential biomarkers of exposure. The diet is a major source of nonoccupational exposure to arsenic, which can be in inorganic and organic forms. Certain types of seafood (e.g., shrimp) contain arsenobetaine, a relatively nontoxic organic form of arsenic. Drinking water contains predominantly inorganic arsenic and can be a significant source of arsenic exposure. Furthermore, although diet is the primary route of general population arsenic exposure, arsenic is also found in the soil and air; thus, inhalation and dermal absorption are additional routes of exposure. Inhalation can be a significant route of occupational exposure to inorganic arsenic (Hughes 2006).

The biomonitoring data for methyl eugenol suggest no sex, racial, and ethnic exposure differences (Barr et al. 2000). Lifestyle differences, such as wine consumption (De Simon et al. 2003) and occupational setting (agricultural use), can also contribute to exposure (Vargas et al. 2000). Because methyl eugenol is found in air, water, and some foods, spices, and oils (Barr et al. 2000; Smith et al. 2002), day-to-day variation in food consumption can significantly affect blood levels of methyl eugenol. Furthermore, because methyl eugenol is rapidly metabolized, sampling and analysis (matrix) strategies can also affect biomonitoring of this chemical. Importantly, the biomonitoring data for methyl eugenol do provide information on total exposure to this chemical, which can be compared with dietary estimates to provide some perspective on the fraction of exposure from food consumption.

Based on the discussions on exposure for each case study, the following questions should be considered when designing, conducting, or interpreting exposure studies in the context of biomonitoring:

- Have the primary sources of exposure been identified?
- Are the pathways/routes of exposure understood?
- Can human exposure be related to animal toxicology studies?
- Is there some understanding of the exposure–dose relationship?
- What is understood about temporality and duration of exposure?

Finally, emerging technologies in molecular biology (e.g., genomics/proteomics, nano-technology) can provide greater insights into environment/gene interactions and can be integrated with traditional biomonitoring data to enhance interpretation of issues such as individual and population differences (Schwartz et al. 2005). For example, the use of molecular approaches may better delineate genetic-based differences in pharmacokinetics that in turn might explain differences in biomonitoring data for different subpopulations. Metabolomics will permit the rapid identification of metabolic differences in populations. Development of nanosensor technologies will greatly facilitate real-time exposure biomonitoring (Balshaw et al. 2005). As these emerging technologies are introduced and applied, serious discussion is warranted and, in fact, under way regarding the ethics associated with the conduct of human pharmacokinetic studies as related to exposure to environmental chemicals. Resolution and guidance on this issue will significantly influence the likelihood that such studies can be conducted and that regulatory agencies would in turn use such data.

## Toxicology/Toxicokinetics

Ideally, sufficient toxicologic data are available in humans and animals to compare results for biomonitoring purposes. In reality, data sets are often limited. For this mini-monograph the critical toxicologic effect(s) associated with each of the case study chemicals is reasonably well defined. Certain limitations do exist, however. For example, in the case of methyl eugenol, the mode of action in animals is not understood, and there are no known human health effects associated with dietary ingestion (Robison and Barr 2006; Schecter et al. 2004). A number of animal toxicity studies with methyl eugenol exist, including single- and multiple-dose toxicokinetic studies. The available data indicate that methyl eugenol undergoes relatively rapid metabolic conversion and excretion (National Toxicology Program 2000; Smith et al. 2002). The rodent bioassay data indicate that methyl eugenol, along with some structural analogs and when administered at high-bolus doses, may cause a shift in metabolism, resulting in the formation of a reactive carbonium ion intermediate. This is associated with liver tumor induction in rodents. Although the oral route of exposure is relevant, use of bolus administration in rodents contrasts with human exposure via dietary ingestion of spices and foods containing methyl eugenol. In addition, low-level dermal exposure may occur after the use of products containing methyl eugenol as a component of natural oils. More data are needed to understand comparative metabolism between animals and humans, the critical metabolite(s), and the mode of action.

The toxicology of inorganic arsenic is well characterized for most end points (Hughes 2006). Inorganic arsenic exposure may result in a number of different toxic effects, including cancer, neurotoxicity, genotoxicity, and cardiovascular toxicity. However, in contrast to most chemical carcinogens, an animal model does not exist for assessing the carcinogenic effects of inorganic arsenic. With this limitation, the mode of action for inorganic arsenic carcinogenesis is not completely understood.

The toxicologic profile of DEP has been fairly well characterized and was recently reviewed [World Health Organization (WHO) 2003]. Although there are limited toxicokinetic data for DEP, it is possible to make some inferences about the toxicokinetics of DEP based on information available for other phthalates (Calafat and McKee 2006). The existing data support the conclusion that there are no substantial differences in metabolism between humans and rodents. Quantitative safety assessments for DEP use one of two no observed adverse effect levels (NOAELs): one from a dietary study (U.S. EPA 1993), and one from a developmental and reproductive toxicity study (WHO 2003). Assessments of exposure (David 2000; Kohn et al. 2000) indicate that the exposures based on biomonitoring data are substantially less than either NOAEL.

Toxicology data indicate that DEHP can induce liver effects in rodents, including changes in liver weight, histological changes, peroxisome proliferation, and tumors (Calafat and McKee 2006). The International Agency for Research on Cancer concluded that there is sufficient evidence of carcinogenicity in animals but insufficient evidence in humans (IARC 2000). There is also evidence that high doses of DEHP can cause developmental and reproductive toxicity. In recent years these effects of DEHP have received more attention than the carcinogenic effects. Quantitative safety assessments for DEHP have used either an NOAEL for noncancer liver effects or the more conservative linear extrapolation methods based on liver tumor induction. Urinary metabolite data indicate that ambient exposures of the U.S. population to DEHP are lower than the reference dose (RfD) established by the U.S. EPA, although use of medical devices may result in much higher exposures to DEHP (Calafat and McKee 2006). However, the use of medical devices entails risk–benefit calculations that make the risk assessments substantially different from those relating to ambient exposures.

The toxicity of PBDE mixtures has been studied in mammals (Birnbaum and Cohen Hubal 2006). This case study highlights some of the considerations that are needed when evaluating the toxicity of mixtures and their individual components. Recently, concerns have been expressed regarding the potential of PBDEs to cause endocrine-related effects (i.e., PBDEs are antiandrogenic and perturb estrogen and progesterone pathways) or developmental toxicity effects based on their qualitative structural similarity to polychlorinated dibenzo-*p*-dioxins and polychlorinated biphenyls (PCBs), although animal toxicity data suggest that PBDEs are not dioxin-like but are more similar to PCBs (Chen et al. 2001). The toxicology of the key primary component molecules, penta-, octa-, and decabromodiphenyl ethers, has been evaluated. Exposure to pentabromodiphenyl ether has been associated with hepatic and endocrine-disruptive effects, developmental reproductive effects, and, of most concern, developmental neurotoxicity. Reproductive effects have been shown in rats, rabbits, and fish. Octabromodiphenyl ether has been shown to cause fetal effects at maternally toxic doses, and contaminating levels of 2,2′,4,4′,5,5′-hexabromodiphenyl ether has also been reported to induce developmental neurotoxicity and perturb several hormonal pathways. Decabromodiphenyl ether (“Deca,” the commercial mixture) is reported to have a relatively low order of toxicity, based on studies conducted to date. There are no long-term toxicity studies for the PBDE mixtures or any of the individual PBDEs. Species differences in PBDE accumulation appear to exist.

The pharmacokinetic properties of PFOS are favorable for using serum PFOS concentration as a measure of internal dose (Butenhoff et al. 2006). Good absorption, lack of known metabolism, distribution primarily in extracellular space, high serum protein binding (albumin and β-lipoproteins), and poor elimination in all species studied combine to establish serum PFOS concentration as an integration of exposures from various sources. In addition, serum PFOS concentrations can be directly associated with effects in toxicology studies.

The acute animal and human toxicities of the organophosphorus pesticides chlorpyrifos and malathion are well understood. As potent inhibitors of the enzyme acetylcholinesterase that breaks down the neurotransmitter acetylcholine, symptoms range from nausea, headaches, and increased salivation to death, depending on the magnitude of exposure. The organophosphorus insecticides are rapidly metabolized and excreted in urine. Many of the urinary metabolites of organophosphorus insecticides are common, preventing the identification of the parent pesticide(s) to which an individual was exposed. Other urinary metabolites are more selective for a given insecticide. Measurements of the intact pesticide in blood are the most specific indicators of exposure to a given organophosphorus pesticide; however, these measurements are complex and may be hampered by their instability in blood.

Based on the discussions of toxicity for each case study, the following questions should be considered when designing, conducting, or interpreting toxicology studies in the context of biomonitoring:

- Are there sufficient toxicology data, including for longer-term exposures?
- Are the routes of exposure in human and toxicology studies comparable?
- Are toxicokinetic data in animals and humans available?
- Are the critical effect(s) and mode of action understood?
- Are the animal data relevant for humans?
- Are matched biologic samples available for both humans and animals so that the results can be compared? Or do sufficient pharmacokinetic data exist to estimate exposure levels in animal studies?

## Epidemiology

Data from epidemiology studies can provide the critical information needed to support the link between human exposure and human health effects. At the September 2004 International Biomonitoring Workshop, the inorganic arsenic case study was used as an example of epidemiology data that provide the initial evidence and link to health effects in humans (Hughes 2006). A number of epidemiology studies have shown that both occupational and environmental exposure to inorganic arsenic elevates the risk of certain cancer types, including bladder, skin, and lung.

Similarly, possible health effects related to organophosphorus pesticide exposures were studied. Apart from the acute toxic effects that have been directly attributable to organophosphorus pesticide exposures in manufacturers, applicators, and suicide victims, low-level environmental exposures to chlorpyrifos (Berkowitz et al. 2004; Perera et al. 2003; Whyatt et al. 2004) or organophosphorus pesticide mixtures (Eskenazi et al. 2004; Young et al. 2005) and birth outcome or neurocognitive effects were evaluated in several studies. However, the exposure measures and birth outcomes associated with the exposures differed (Needham 2005).

For the other case study chemicals, there are limited or no epidemiology data. For example, for methyl eugenol and PBDEs, there are no known epidemiology studies; however, extensive human dietary exposure data exist (Robison and Barr 2006).

Currently, evidence of human health effects associated with exposure to DEP or DEHP is limited (Calafat and McKee 2006). No epidemiology data for DEHP exist to suggest a carcinogenic effect, and several recent reviews question whether the existing liver tumor data in animals are relevant for humans (IARC 2000; Klaunig et al. 2003). The present concern for DEHP exposure is the potential for developmental and reproductive effects. In several human studies, possible associations between phthalates, including DEP and DEHP, and altered semen quality in adults as well as shortened anogenital distance in baby boys, and other health end points have been explored (Calafat and McKee 2006). Because some of these studies suggest potential adverse health effects of phthalates, further studies with larger populations are recommended.

No large-scale epidemiology studies exist for PBDEs or PFOS (Birnbaum and Cohen Hubal 2006; Butenhoff et al. 2006). Very limited human data are available to suggest any human health effects associated with PBDE exposure. For PFOS, analyses of occupationally exposed workers are available, including one cohort mortality study (Alexander et al. 2003) that was followed up by a worker health survey (Alexander 2004). There is some indication that a higher risk of bladder cancer mortality exists in some of the highly exposed workers, but the limited size of the study population precluded a conclusive exposure–response analysis (Butenhoff et al. 2006).

The case studies illustrate that, in many instances, there is an absence of large-scale epidemiology studies with sufficient statistical power to detect associations between human exposure and health effects identified in animal toxicology studies. In addition, interpretation of epidemiologic studies is complicated by the limited ability to accurately determine dosimetry, exposure duration, and patterns of exposure. Defining the potential confounding factors for each chemical is important because epidemiology studies are often designed to evaluate a specific association between chemical exposure and a known health effect(s).

Several factors should be considered when designing, conducting, or interpreting epidemiologic studies that seek to define associations between specific exposures and specific human health effects (or their absence), particularly in the context of biomonitoring. As stated previously, careful definition of the link being assessed, as well as the question being asked, is critical.

- Are criteria for making reasonable inferences of association and causation supported? The Bradford Hill criteria (Federal Focus 1996), used successfully in the context of establishing causality in many epidemiology studies, may have similar utility in biomonitoring studies. These criteria consist of the following basic characteristics: the strength, specificity, and consistency of the association; the temporality and duration of exposure; the biologic gradient or the relationship between the dose and the response; the effects of the removal of the suggested cause; the biologic plausibility of the association; and the coherence between the association and other findings.
- Has an adverse health effect been demonstrated in humans?
- Is there information regarding the mode of action for the agent producing this health effect?
- Are there health effects observed in populations exposed to the agent of concern? (Note that some characterization of the health effects observed in populations exposed to the agent of concern is needed to design a new epidemiologic study that is focused on disease end points. Furthermore, these health effects must be known before biomarkers are used to identify population exposures and assess risk.)
- Are any toxicokinetic and/or toxicodynamic genetic polymorphisms known to modify risk and define susceptible populations?

## Risk Assessment/Risk Management

As noted previously, biomonitoring data can be used in multiple ways (e.g., trend analysis, exposure assessment, dose reconstruction), and supporting data, such as those necessary for risk assessment purposes, are not always needed. When biomonitoring data are used for these non-risk assessment purposes, the uncertainty associated with their intended use(s) should be acknowledged and communicated. In terms of risk assessment and risk management (e.g., refining remediation efforts), biomonitoring data have the potential to be a valuable tool.

Given the increased sensitivity of analytical methods, simple detection of a chemical in biologic samples such as blood, urine, breast milk, or body fat should not be confused with or equated to increased risk. Exposure information must be carefully evaluated against all relevant toxicology data and any human epidemiology data. In addition, the relevance of the toxicology data to humans should be considered. There are, based on epidemiology data, biologically plausible statistical associations that, taken together with animal and other toxicologic data, imply causation between exposure and health effects for inorganic arsenic and organophosphorus pesticides. Evidence suggesting human health effects associated with exposure to DEP, DEHP, methyl eugenol, and PFOS is limited and/or restricted to statistical associations.

For the phthalates, methyl eugenol, and PBDEs, comparison of exposures based on the NHANES biomonitoring data that represent aggregate exposures to all sources offers suggestive evidence that human exposure in the general population is lower than NOAELs or RfDs derived from animal toxicology studies (CDC 2001, 2003, 2005). However, trends in internal concentrations of certain compounds detected via biomonitoring (e.g., rapidly rising PBDE levels in the United States), as well as the impact on vulnerable populations (i.e., highly exposed or highly susceptible to effects), should also be considered in assessing risk and making risk management decisions. Emerging data on human blood levels of PBDEs for highly exposed individuals show that there is no margin of exposure based on several published animal studies of developmental or neurologic toxicity (Birnbaum and Cohen Hubal 2006).

Based on the discussions of risk assessment and risk management for each case study, the following questions should be considered when designing, conducting, or interpreting studies for biomonitoring purposes:

- Are there sufficient and relevant toxicology data?
- Is there a relationship between the biomarker of exposure and a known human health effect?
- Are there pharmacokinetic data that can be useful in the risk assessment?
- If applicable, is there evidence that remediation efforts are working?

## Research Needs/Data Gaps

Meaningful interpretation of existing and future biomonitoring data will require rigorous, scientific approaches to data collection, analysis, interpretation, and application. Thus, biomonitoring data can provide much-needed information on exposure to a variety of environmental chemicals. However, investigators must define and communicate the question to be addressed in any given biomonitoring study. For example, the data required for the assessment and interpretation of exposure trends may be different from those necessary for the assessment of health risk. Nonetheless, the lack of seemingly critical pieces of data for proper interpretation of biomonitoring data does not render the biomonitoring data unusable. Rather, the existing data gaps add to the uncertainty of the interpretation of the biomonitoring data. Filling these critical data gaps is essential to reduce these uncertainties in interpretation, thus providing the most reliable data for public health decisions.

**Figure 1:**
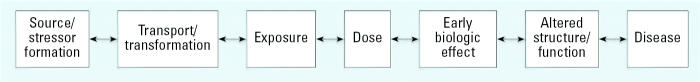
The environmental public health continuum (EPHC).

The authors of this mini-monograph call for research activities in the following areas to advance scientific understanding and application of biomonitoring data in its various contexts:

- Improve the understanding of the predictive relationships/linkages between measures of exposure, dose, and effect. Such insight would allow the development of an interpretation strategy and specific criteria for moving from any point on the EPHC (Figure 1) toward either the “exposure” or “effects” sides.
- Emphasize biomarker validation and precision. For analytical measurements, conduct interlaboratory comparison trials.
- Characterize a baseline for biomarkers, and apply statistical methods to assess temporal departures from the baseline.
- Improve understanding of the origin of the biomarker and its relationship to the disease process and/or individual, multiple, and exogenous or endogenous exposure. Establish a database of biomarker disease associations, including null and negative studies.
- Improve study design to better assess intra-and interindividual variability related to measures of exposure, dose, metabolism, and effects that would influence the likelihood of observing predictive relationships between these variables and aid in identifying subpopulations that might be at greater risk. Such data would also clarify the relevance of biomarkers for the target tissues of certain organs.
- Apply new technologies such as gene expression, proteomics, and protein activity profiling, both in terms of development of potential new biomarkers and as screening tools for identifying candidates for biomonitoring.

The questions and considerations identified in this article for designing, conducting, or interpreting studies for biomonitoring purposes are intended as guidance only. The authors acknowledge that no individual study can address every question. It is recommended, however, that future studies be designed with some or all of these considerations in mind to maximize application and interpretation of biomonitoring data for human health risk assessment.

Government, academic, and industry scientists who are committed to identifying data needs and exploring research programs through the HESI consensus-building process will report progress and technical advancements in biomonitoring in future publications.

## Figures and Tables

**Figure 2:**
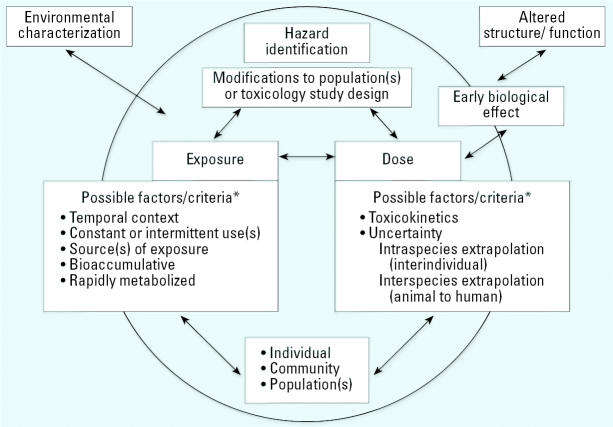
Focus area for the September 2004 International Biomonitoring Workshop. *Factors/criteria discussed at the workshop.

## References

[b1-ehp0114-001755] AHS (Agricultural Health Study) 2005. Home Page. Available: http://www.aghealth.org/results.html [accessed 9 April 2006].

[b2-ehp0114-001755] Alexander BH 2004. Bladder Cancer in Perfluorooctanesulfonyl Fluoride Manufacturing Workers. U.S. EPA Docket AR-226-1958. Washington, DC:U.S. Environmental Protection Agency.

[b3-ehp0114-001755] Alexander BH, Olsen GW, Burris JM, Mandel JH, Mandel JS (2003). Mortality of employees of a perfluorooctanesulfonyl fluoride manufacturing facility. Occup Environ Med.

[b4-ehp0114-001755] Api AM (2001). Toxicological profile of diethyl phthalate: a vehicle for fragrance and cosmetic ingredients. Food Chem Toxicol.

[b5-ehp0114-001755] Balshaw DM, Philbert M, Suk WA (2005). Research strategies for safety evaluation of nanomaterials. Part III: Nanoscale technologies for assessing risk and improving public health. Toxicol Sci.

[b6-ehp0114-001755] Barr DB, Allen R, Olsson AO, Bravo R, Caltabiano LM, Montesano A (2005). Concentrations of selective metabolites of organophosphorus pesticides in the United States population. Environ Res.

[b7-ehp0114-001755] Barr DB, Angerer J (2006). Potential uses of biomonitoring data: a case study using the organophosphorus pesticides chlorpyrifos and malathion. Environ Health Perspect.

[b8-ehp0114-001755] Barr DB, Barr JR, Bailey SL, Lapeza CR, Beeson MD, Caudill SP (2000). Levels of methyl eugenol in a subset of adults in the general U.S. population as determined by high resolution mass spectrometry. Environ Health Perspect.

[b9-ehp0114-001755] Barr DB, Bravo R, Weerasekera G, Caltabiano LM, Whitehead RD, Olsson AO (2004). Concentrations of dialkyl phosphate metabolites of organophosphorus pesticides in the U.S. population. Environ Health Perspect.

[b10-ehp0114-001755] Berkowitz GS, Wetmur JG, Birman-Deych E, Obel J, Lapinski RH, Godbold JH (2004). *In utero* pesticide exposure, maternal paraoxonase activity, and head circumference. Environ Health Perspect.

[b11-ehp0114-001755] Birnbaum LS, Cohen Hubal EA (2006). Polybrominated diphenyl ethers: a case study for using biomonitoring data to address risk assessment questions. Environ Health Perspect.

[b12-ehp0114-001755] Butenhoff JL, Olsen GW, Pfahles-Hutchens A (2006). The applicability of biomonitoring data for perfluorooctanesulfonate to the environmental public health continuum. Environ Health Perspect.

[b13-ehp0114-001755] Calafat AM, McKee RH (2006). Integrating biomonitoring exposure data into the risk assessment process: phthalates (diethyl phthalate and di[2-ethylhexyl] phthalate) as a case study. Environ Health Perspect.

[b14-ehp0114-001755] CDC 2001. National Report on Human Exposure to Environmental Chemicals. Atlanta:Centers for Disease Control and Prevention. Available: http://www.cdc.gov/nchs/about/major/nhanes/datalink.htm [accessed 9 April 2006].

[b15-ehp0114-001755] CDC 2003. Second National Report on Human Exposure to Environmental Chemicals. Atlanta:Centers for Disease Control and Prevention. Available: http://www.cdc.gov/nchs/about/major/nhanes/datalink.htm [accessed 9 April 2006].

[b16-ehp0114-001755] CDC 2004. Environmental Public Health Tracking Program. Atlanta:Centers for Disease Control and Prevention. Available: http://www.cdc.gov/nceh/tracking/ [accessed 9 April 2006].

[b17-ehp0114-001755] CDC 2005. Third National Report on Human Exposure to Environmental Chemicals. Atlanta:Centers for Disease Control and Prevention. Available: http://www.cdc.gov/exposurereport/ [accessed 9 April 2006].

[b18-ehp0114-001755] Chen G, Konstantinov AD, Chittim BG, Joyce EM, Bols NC, Bunce NJ (2001). Synthesis of polybrominated diphenyl ethers and their capacity to induce CYP1A by the Ah receptor mediated pathway. Environ Sci Technol.

[b19-ehp0114-001755] CLIA 1988. Clinical Laboratory Improvement Amendments of 1988. 42 Code of Federal Regulations Part 493, Laboratory Requirements. Available: http://www.phppo.cdc.gov/clia/regs/toc.aspx [accessed 9 April 2006].

[b20-ehp0114-001755] Concha G, Nermell B, Vahter M (1998). Metabolism of inorganic arsenic in children with chronic high arsenic exposure in Northern Argentina. Environ Health Perspect.

[b21-ehp0114-001755] David RM (2000). Exposure to phthalate esters [Letter]. Environ Health Perspect.

[b22-ehp0114-001755] De Simon BF, Cadahia E, Jalocha J (2003). Volatile compounds in a Spanish red wine aged in barrels made of Spanish, French, and American oak wood. J Agric Food Chem.

[b23-ehp0114-001755] Doerrer ND, Holsapple MP (2004). Integration of biomonitoring exposure data into the risk assessment process. Risk Pol Rep.

[b24-ehp0114-001755] EC (European Commission) 2004a. Baseline Report on “Biomonitoring of Children” in the Framework of the European Environment and Health Strategy (COM(2003)338 final). Technical Working Group on Integrated Monitoring, Subgroup on Biomonitoring of Children. Munich:European Commission. Available: http://www.brussels-conference.org/Download/baseline_report/BR_Biomonitoring_final.pdf [accessed 9 April 2006].

[b25-ehp0114-001755] EC (European Commission) 2004b. European Human Biomonitoring Homepage of the Implementation Group and ESBIO. Available: http://www.eu-biomonitoring.org/ [accessed 14 April 2006].

[b26-ehp0114-001755] ECETOC 2005. Guidance for the Interpretation of Biomonitoring Data. Document No. 44. Brussels:European Centre for Ecotoxicology and Toxicology of Chemicals.

[b27-ehp0114-001755] Eskenazi B, Harley K, Bradman A, Weltzien E, Jewell NP, Barr DB (2004). Association of *in utero* organophosphate pesticide exposure and fetal growth and length of gestation in an agricultural population. Environ Health Perspect.

[b28-ehp0114-001755] Federal Focus 1996. Principles for Evaluating Epidemiologic Data in Regulatory Risk Assessment. Appendix B. Washington, DC:Federal Focus, Inc.

[b29-ehp0114-001755] Hughes MF (2006). Biomarkers of exposure: a case study with inorganic arsenic. Environ Health Perspect.

[b30-ehp0114-001755] IARC (International Agency for Research on Cancer) (2000). Some Industrial Chemicals. IARC Monogr Eval Carcinog Risks Hum.

[b31-ehp0114-001755] Klaunig JE, Babich MA, Baetcke KP, Cook JC, Corton JC, David RM (2003). PPAR alpha agonist-induced rodent tumors: modes of action and human relevance. Crit Rev Toxicol.

[b32-ehp0114-001755] Kohn MC, Parham F, Masten SA, Portier CJ, Shelby MD, Brock JW (2000). Human exposure estimates for phthalates [Letter]. Environ Health Perspect.

[b33-ehp0114-001755] NCS (National Children’s Study) 2005. Home Page. Available: http://nationalchildrensstudy.gov/ [accessed 6 April 2006].

[b34-ehp0114-001755] Needham LL (2005). Assessing exposure to organophosphorus pesticides by biomonitoring in epidemiologic studies of birth outcomes. Environ Health Perspect.

[b35-ehp0114-001755] NIEHS 2003. Centers for Children’s Environmental Health and Disease Prevention Research. Research Triangle Park, NC: National Institute of Environmental Health Sciences. Available: http://www.niehs.nih.gov/translat/children/children.htm [accessed 9 April 2006].

[b36-ehp0114-001755] NRC (National Research Council) 2004. Human Biomonitoring for Environmental Chemicals. National Research Council Committee on Human Biomonitoring for Environmental Toxicants. Washington, DC:National Academies Press. Available: http://www.nap.edu/catalog/11700.html [accessed 9 April 2006].

[b37-ehp0114-001755] National Toxicology Program 2000. Toxicology and Carcinogenesis Studies of Methyleugenol (CAS no. 93-15-2) in F344/N Rats and B6C3F_1_ Mice (Gavage Studies). Technical Report 491. Research Triangle Park, NC:National Toxicology Program. 12563349

[b38-ehp0114-001755] Perera FP, Rauh V, Tsai WY, Kinney P, Camann D, Barr D (2003). Effects of transplacental exposure to environmental pollutants on birth outcomes in a multiethnic population. Environ Health Perspect.

[b39-ehp0114-001755] Robison SH, Barr DB (2006). Use of biomonitoring data to evaluate methyl eugenol exposure. Environ Health Perspect.

[b40-ehp0114-001755] Schecter A, Lucier GW, Cunningham ML, Abdo KM, Blumenthal G, Silver AG (2004). Human consumption of methyl-eugenol and its elimination from serum. Environ Health Perspect.

[b41-ehp0114-001755] Schwartz DA, Weis B, Wilson SH (2005). The need for exposure health sciences. Environ Health Perspect.

[b42-ehp0114-001755] Sexton K, Needham LL, Pirkle JL (2004). Human biomonitoring of environmental chemicals. Am Sci.

[b43-ehp0114-001755] Smith R, Adams T, Doull J, Feron V, Goodman J, Marnett L (2002). Safety assessment of allylalkoxybenzene derivatives used in flavoring substances—methyl eugenol and estragole. Food Chem Toxicol.

[b44-ehp0114-001755] Toniolo P, Boffetta P, Shuker DEG, Rothman N, Hulka B, Pearce N (1997). Application of Biomarkers in Cancer Epidemiology—Workshop Report. IARC Sci Publ.

[b45-ehp0114-001755] University of Minnesota 2004. Farm Family Exposure Study. Available: http://www.farmfamilyexposure.org/ [accessed 9 April 2006].

[b46-ehp0114-001755] U.S. EPA 1993. Diethyl Phthalate (CASRN 84-66-2). Washington, DC:U.S. Environmental Protection Agency. Available: http://www.epa.gov/iris/subst/0226.htm [accessed 9 April 2006].

[b47-ehp0114-001755] U.S. EPA 2004. National Human Exposure Assessment Survey (NHEXAS). Washington, DC:U.S. Environmental Protection Agency. Available: http://www.epa.gov/nerl/research/2002/g8-2.html [accessed 9 April 2006].

[b48-ehp0114-001755] Vargas RI, Stark JD, Kido MH, Ketter HM, Whitehand LC (2000). Methyl eugenol and cue-lure traps for suppression of male oriental fruit flies and melon flies (Diptera: Tephritidae) in Hawaii: effects of lure mixtures and weathering. J Econ Entomol.

[b49-ehp0114-001755] WHO 2003. Diethyl Phthalate. Concise International Chemical Assessment Document (CICAD) 52. Geneva:World Health Organization. Available: http://www.who.int/ipcs/publications/cicad/en/cicad52.pdf [accessed 9 April 2006].

[b50-ehp0114-001755] Whyatt RM, Rauh V, Barr DB, Camann DE, Andrews HF, Garfinkel R (2004). Prenatal insecticide exposures and birth weight and length among an urban minority cohort. Environ Health Perspect.

[b51-ehp0114-001755] WWF 2004. Bad Blood? A Survey of Chemicals in the Blood of European Ministers. Brussels:World Wildlife Fund. Available: http://worldwildlife.org/toxics/pubs/badblood.pdf [accessed 9 April 2006].

[b52-ehp0114-001755] Young JG, Eskenazi B, Gladstone EA, Bradman A, Pedersen L, Johnson C (2005). Association between *in utero* organophosphorus pesticide exposure and abnormal reflexes in neonates. Neurotoxicology.

